# Molecular cloning of porcine Siglec-3, Siglec-5 and Siglec-10, and identification of Siglec-10 as an alternative receptor for porcine reproductive and respiratory syndrome virus (PRRSV)

**DOI:** 10.1099/jgv.0.000859

**Published:** 2017-07-26

**Authors:** Jiexiong Xie, Isaura Christiaens, Bo Yang, Wander Van Breedam, Tingting Cui, Hans J. Nauwynck

**Affiliations:** ^1^​ Laboratory of Virology, Faculty of Veterinary Medicine, Ghent University, Salisburylaan 133, B-9820 Merelbeke, Belgium; ^2^​ Unit for Medical Biotechnology, Medical Biotechnology Center, VIB, Technologiepark 927, B-9052 Ghent, Belgium

**Keywords:** PRRSV, siglecs, receptor

## Abstract

In recent years, several entry mediators have been characterized for porcine reproductive and respiratory syndrome virus (PRRSV). Porcine sialoadhesin [pSn, also known as sialic acid-binding immunoglobulin-type lectin (Siglec-1)] and porcine CD163 (pCD163) have been identified as the most important host entry mediators that can fully coordinate PRRSV infection into macrophages. However, recent isolates have not only shown a tropism for sialoadhesin-positive cells, but also for sialoadhesin-negative cells. This observation might be partly explained by the existence of additional receptors that can support PRRSV binding and entry. In the search for new receptors, recently identified porcine Siglecs (Siglec-3, Siglec-5 and Siglec-10), members of the same family as sialoadhesin, were cloned and characterized. Only Siglec-10 was able to significantly improve PRRSV infection and production in a CD163-transfected cell line. Compared with sialoadhesin, Siglec-10 performed equally effectively as a receptor for PRRSV type 2 strain MN-184, but it was less capable of supporting infection with PRRSV type 1 strain LV (Lelystad virus). Siglec-10 was demonstrated to be involved in the endocytosis of PRRSV, confirming the important role of Siglec-10 in the entry process of PRRSV. In conclusion, it can be stated that PRRSV may use several Siglecs to enter macrophages, which may explain the strain differences in the pathogenesis.

## Abbreviations

CPE, cytopathic effect; Endo H, endoglycosidase H; IL-1, interleukin 1; LV, Lelystad virus; MAG, myelin-associated glycoprotein; NF-κB, nuclear factor kappa-light-chain-enhancer of activated B cells; pCD163, porcine Cluster of Differentiation 163; PK-15, porcine kidney cells; PNGase F, peptide -N-glycosidase F; PRRSV, porcine reproductive and respiratory syndrome virus; pSn, Porcine sialoadhesin; ROI, region of interest; Siglec, sialic acid-binding immunoglobulin-type lectins; TNF-α, tumor necrosis factor alpha.

## Introduction

Porcine reproductive and respiratory syndrome (PRRS) is one of the most economically devastating diseases in the pig industry [1]. The disease is associated with respiratory disorders in piglets and reproductive problems in sows. PRRS virus (PRRSV), the etiology of PRRS, is a single-stranded enveloped RNA virus belonging to the order *Nidovirales*, family *Arteriviridae* and genus *Arterivirus* [2]. Currently, two types of PRRSV have been reported, the European type (known as type 1) and the North American type (known as type 2), with huge genetic variability between and within each genotype [3, 4].

PRRSV has a narrow cell tropism for cells both *in vivo* and *in vitro*. Differentiated macrophages are the main target cells, with specific entry mediators determining whether cells are permissive to PRRSV infection. Up till now, several receptors such as heparan sulfate, porcine sialoadhesin (pSn), CD163, CD151, vimentin and DC-SIGN, have been identified as entry mediators for PRRSV [5–7]. The interaction of heparan sulfate with the GP5/M protein complex mediates the binding of the virus [8]. pSn, also known as porcine sialic acid-binding immunoglobulin-type lectin-1 (Siglec-1) is associated with attachment and internalization in a sialic acid-dependent manner [6, 9]. Porcine CD163 (pCD163) interacts with GP2a and/or GP4 to mediate disassembly and genome release [10, 11]. pSn and CD163 have been identified as the most important host receptors that facilitate the infection of PRRSV into alveolar macrophages [5, 6, 12]. However, recent research has reported that the expression of Siglec-1 in pigs is not required for infection with the PRRSV North American strain KS-06 [13]. Meanwhile, research from our laboratory has shown that different isolates exhibit variable cell tropism. Certain emerging isolates, such as Lena from Belarus (type 1, subtype 3, 2006, accession number: JF802085), 13V091 (type 1, subtype 1, 2013, accession number: KT159248) from Belgium and MN-184 from the USA (type 2, 2002, accession number: DQ176019) have not only shown a strong tropism for sialoadhesin-positive macrophages, but also for sialoadhesin-negative macrophages. This observation suggests that additional receptors that can replace the role of Siglec-1 as the receptor enabling PRRSV replication exist [14, 15].

At present, two primary groups of Siglecs have been identified in humans. One group comprising Siglec-1, CD22 (Siglec-2), myelin-associated glycoprotein (MAG; Siglec-4) and Siglec-15, are relatively well conserved in mammals. The other group, known as the CD33-related Siglecs, consists of CD33 (Siglec-3), Siglec-5 to -13, Siglec-14 and Siglec-16. The CD33-related group is evolving rapidly and exhibits differences in composition between mammalian species [16, 17]. In humans, CD33-related Siglecs interact with several sialylated pathogens, such as *Campylobacter jejuni, Neisseria meningitides,* group B streptococcus*, Trypanosoma cruzi* and human immunodeficiency virus (HIV) [18, 19]. Siglec-7 has been reported to interact with the gp120 of HIV-1 and to facilitate the infection of CD4^+^ T cells and macrophages [19]. However, up till now few of the Siglecs have been identified in pigs. Recently, Siglec-3, Siglec-5 and Siglec-10 were cloned and characterized in pigs [20–22]. Since Siglec-1 does not seem to be the only receptor for PRRSV, and the Siglecs reported in humans are frequently used as receptors for various pathogens, we investigated the functions of the already characterized porcine Siglecs and aimed to identify Siglecs that may have similar functions to Siglec-1.

## Results

### Amino acid sequence, structure and expression analysis of Siglecs

**Fig. 1.**
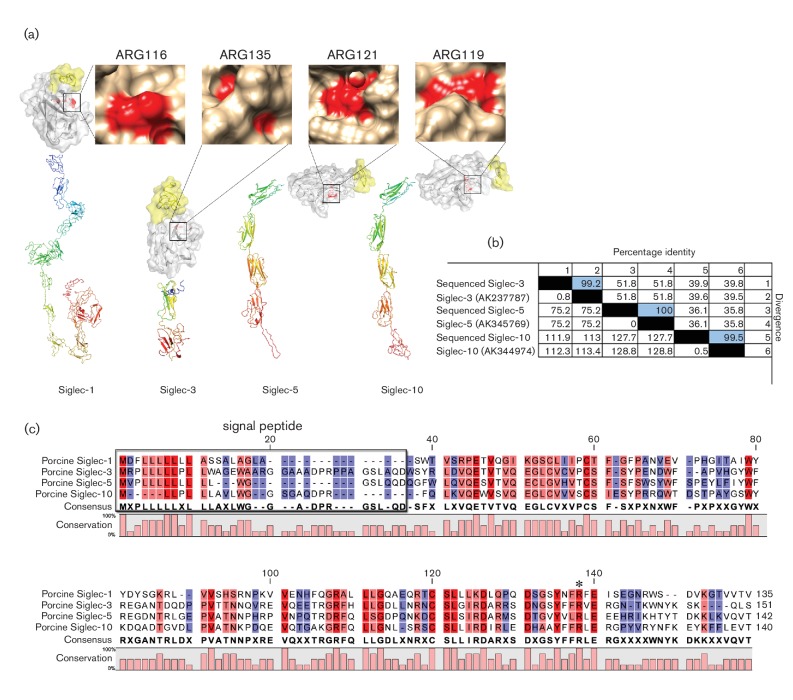
Amino acid sequence and structure analysis of Siglec-1, Siglec-3, Siglec-5 and Siglec-10. (a) Spatial structure of Siglec-1,3, Siglec-5 and Siglec-10. Protein structure was predicted by I-TASSER (http://zhanglab.ccmb.med.umich.edu/I-TASSER/) and analysed by PyMOL (version 6.6). The yellow portion represents the signal peptide; the white portion represents the V-type Ig domain; and the red dots are the predicted sialic acid-binding site. Different numbers of C-set domains are shown just under the V-type Ig domain. The surface of the predicted sialic acid-binding sites was expanded and the numbers represent the amino acid position of the predicted sialic acid-binding sites. (b) Amino acid sequence homology analysis. The homology of the full-length Siglecs obtained in this study was compared with the sequences that have been reported using MegAlign (DNAstar). Highlighted numbers represent the similarity between the Siglecs obtained in this study with the reported ones. (c) Amino acid comparison of V-type Ig domain. The V-type Ig amino acid sequences of Siglec-1, -3, -5 and -10 were compared using CLC sequence viewer (version 6.8.1). The predicted sialic acid-binding site is indicated with an asterisk.

To better understand Siglec-3, Siglec-5 and Siglec-10, their amino acid sequences were deduced and their structure was predicted with I-TASSER and by PyMOL V6.6. As expected, all Siglecs showed a similar structure to Siglec-1, which includes one V-type and different numbers of C2-type Ig-like domains, a transmembrane domain and a cytoplasmic tail. As shown in Fig. 1(a), Siglec-1 has 16 C2-type Ig-like domains, whereas Siglec-3 has only one C2-type Ig-like domain, and Siglec-5 and Siglec-10 have three C2-type Ig-like domains. The V-type Ig domain is indicated in white, the signal peptide is indicated in yellow and the sialic acid-binding site is indicated in red. The V-type Ig domains of the Siglecs shared a high amino acid homology, as shown in Fig. 1(c). The conserved sites are coloured in red. The predicted sialic acid-binding sites, indicated with a star, were well conserved among these Siglecs. The sequence of the Siglecs obtained in this study showed a high amino acid similarity with the Siglec-3 (accession number: AK237787), Siglec-5 (accession number: AK345769) and Siglec-10 (accession number: AK344974) sequences reported previously by Alvarez *et al.* [20] and [22] (ranging from 99.2–100 %) (Fig. 1b).

**Fig. 2.**
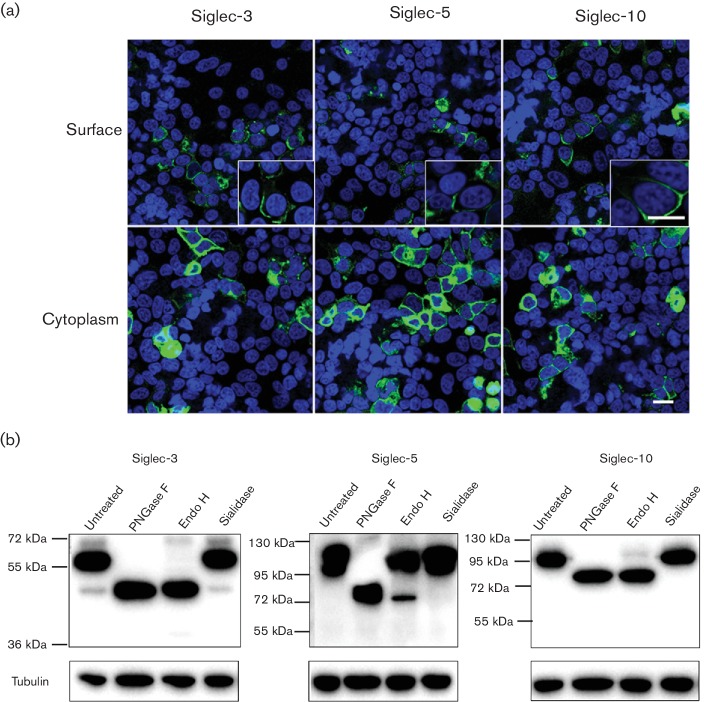
Expression analysis of Siglec-3, Siglec-5 and Siglec-10 using IFA and Western blotting. (a) PK-15 cells were transfected with Siglec-3-, Siglec-5- and Siglec-10-encoding vector. Twenty-four hours post-transfection, the cells were fixed with 4 % PF and permeabilized with Triton X-100 for cytoplasmic staining, or not permeabilized for surface staining. IFA was performed using V5-specific antibody (Siglec; green) and Hoechst 33 342 (nuclei; blue). Scale bar: 25 µm (b) Western blot identification. HEK-293T cells were transfected with Siglec-3, Siglec-5 and Siglec-10. Twenty-four hours post-transfection, the cells were collected and lysed with lysis buffer. Afterwards, cellular lysates were treated or mock-treated with sialidase, EndoH or PNGase F and analysed by SDS-PAGE and Western blot. Primary antibody V5-specific mAb (GenScript; A00641) and secondary peroxidase-labelled goat anti-mouse IgG antibodies (Dako) were used for immunoblotting. For the detection of tubulin, an HRP-conjugated anti-alpha tubulin monoclonal antibody (Abcam; ab40742) was used.

After the successful construction of the porcine Siglec-3-, Siglec-5- and Siglec-10-encoding plasmids, the expression of the Siglecs was examined using immunofluorescence staining and Western blot. PK-15 cells were transfected with the Siglec-encoding constructs. Siglec-3, Siglec-5 and Siglec-10 were successfully expressed both in the cytoplasm and at the surface of the cells (Fig. 2a). To further verify the correct expression of these Siglecs, a Western blot assay was performed. Based on the amino acid sequence and the size of the tag, the estimated sizes of Siglec-3, Siglec-5 and Siglec-10 should be approximately 41 kD, 64 kD and 71 kD, respectively. The obtained sizes for Siglec-3, Siglec-5 and Siglec-10 were approximately 60 kD, 120 kD and 95 kD, respectively, which is larger than the predicted ones (Fig. 2b). To find out if the discrepancy between the predicted sizes and the observed sizes was due to post-translational modification(s) such as glycosylation, a deglycosylation assay was performed. Cell lysates were treated with different glycosidases and analysed by reducing SDS-PAGE and Western blot (Fig. 2b). Treatment of cell lysates with N-glycosidase F (PNGase F), which removes all types of N-linked glycans, resulted in a single species for each Siglec, whose size was in accordance with the predicted size. Treatment with endoglycosidase H (Endo H), which removes high mannose and some hybrid types of N-linked carbohydrates, resulted in two species for all Siglecs: a larger EndoH-resistant form and a smaller EndoH-sensitive form. This implies that the proteins were partly Golgi-processed and contained Endo H-resistant complex oligosaccharides. Finally, cell lysates were treated with *Vibrio cholerae* sialidase to remove sialic acids in the α2–3, α2–6 or α2–8 configuration. Sialidase treatment did not increase the electrophoretic mobility of the proteins. The results of the sialidase treatments indicated that these proteins carry both high mannose and complex type N-glycans capped with few or no sialic acids.

### Transfected cells expressing Siglec-10 exhibit red blood-binding capacity in a sialic acid-dependent manner

**Fig. 3.**
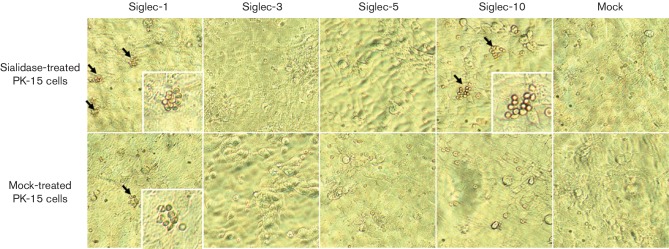
Analysis of the sialic acid-binding capacity of Siglec-1, Siglec-3, Siglec-5 and Siglec-10 by red blood cell binding assay. PK-15 cells that had been transiently transfected with the Siglec-1-, Siglec-3-, Siglec-5- and Siglec-10-encoding vectors were pre-treated with sialidase or mock-treated and incubated with human erythrocytes. Subsequently, the cells were washed and erythrocyte binding was evaluated via light microscopy. The black arrows indicate typical sialic acid-dependent erythrocyte binding. Red blood cell binding was only observed on cells expressing Siglec-1 and Siglec-10.

The sialic acid-binding capacity of the different Siglecs was analysed using a red blood cell binding assay. Only Siglec-1- and Siglec-10-transfected cells showed binding of red blood cells, as depicted with black arrows in (Fig. 3). Siglec-1-expressing PK-15 cells that were not treated with sialidase were still able to bind red blood cells, although to a lesser extent than cells treated with sialidase. This indicates the existence of cis-acting sialic acids for both Siglec-1 and Siglec-10 [23]. When red blood cells were treated with sialidase, no binding was observed for any of these Siglecs (data not shown). In conclusion, it can be stated that Siglec-1 and Siglec-10 are sialic acid-binding lectins that show red blood cell-binding capability in a sialic acid-dependent manner [21, 23].

### Siglec-10 can increase the infection and production of PRRSV in a non-permissive cell line in combination with CD163

**Fig. 4.**
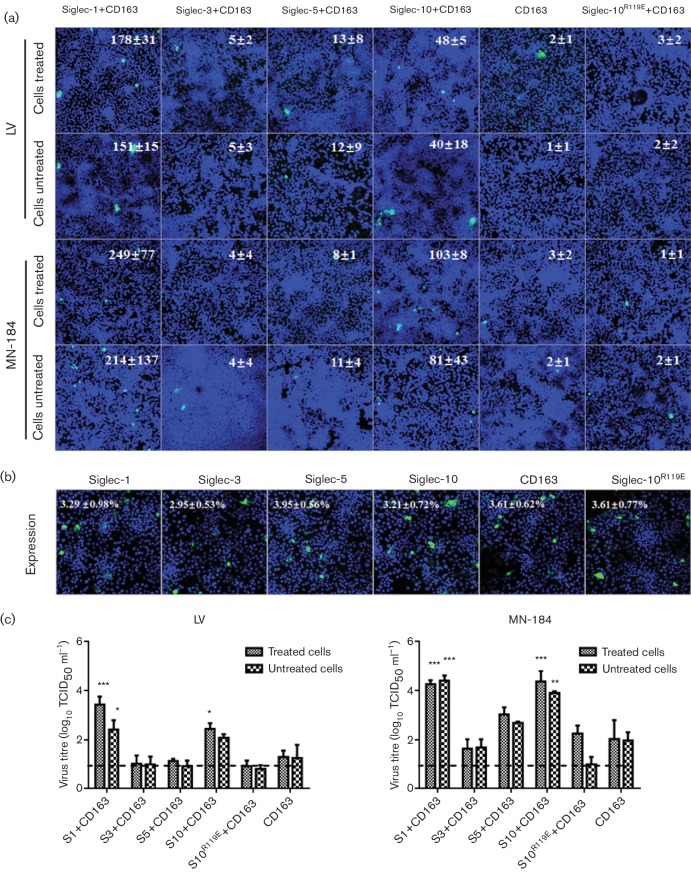
Virus production for the different transfected PK-15 cell groups 24 h after infection. PK-15 cells were transiently transfected with a pCD163-encoding vector in combination with a Siglec-1, Siglec-3, Siglec-5, Siglec-10 or control vector, and 48 h after transfection the cells were treated or not treated with sialidase for 1 h and inoculated with PRRSV LV or MN-184 for 1 h. Twenty-four hours post-infection, the cells were fixed and stained for infection and expression of the different Siglecs and CD163. (a) Immunofluorescence staining of infected cells with mAb 13E2 (against PRRSV nucleocapsid protein; green) [26, 43] and Hoechst 33 342 (nuclei; blue). The absolute number of infected cells for each condition was determined and displayed in the images as the mean ± SEM of three independent experiments. Scale bar: 50 µm. (b) Expression analysis of the different Siglecs using fluorescence microscopy. PK-15 cells were fixed, permeabilized and stained with V5-specific mAb (green) and Hoechst 33 342 (nuclei; blue). The absolute number of transfected cells was determined for each condition and is displayed in the images as the mean ± SEM of three independent experiments. Scale bar: 50 µm. (c) To evaluate virus production, the cell supernatants collected at 24 h p.i. were titrated and the results are displayed in the graphs. The CD163/Siglec double-transfected groups that were significantly different from the CD163 single-transfected group are represented as *P<0.05; **P<0.01 and ***P<0.001.

To further analyse the function of Siglec-10, the Siglec-encoding constructs were co-transfected with CD163-encoding constructs. Forty-eight hours post-transfection, cells were treated with sialidase or mock-treated and inoculated with PRRSV [Lelystad virus (LV)] or PRRSV MN-184. Twenty-four hours post-infection, the cells were fixed and stained for the PRRSV nucleocapsid protein and the supernatants were collected for virus titration. As shown in Fig. 4(a), more virus-positive cells were observed in cells expressing Siglec-1 or Siglec-10 in combination with CD163 than in cells that only expressed CD163. The expression of Siglec-3 and Siglec-5 in combination with CD163 did not significantly increase infection compared to cells that only expressed CD163. Similar results were found for virus titration. Significantly higher virus production was observed in cells expressing Siglec-1 and Siglec-10 together with CD163 compared with cells that only expressed CD163 (Fig. 4b). Neuraminidase treatment of Siglec-1- and Siglec-10-transfected cells significantly increased virus production in the cells infected with MN-184 and LV compared to the mock-treated group (*P*<0.01). Siglec-1 and Siglec-10 also increased virus production in the untreated cells. This increase was significant for MN-184 (*P*<0.01) but not for LV. These results show that in combination with CD163, Siglec-10 is able to improve the infection of PRRSV for both LV and MN-184, and this enhancement is more pronounced for MN-184. Neuraminidase treatment of the target cells increased infection considerably.

Human Siglec-10 and other Siglec family members exhibit a higly conserved predicted sialic acid-binding site. To further investigate the importance of the Siglec-10 sialic acid-binding activity, mutagenesis was performed. A mutation of R at position 119 into E was introduced in the predicted sialic acid-binding site, which gave rise to a mutant, Siglec-10^R119E^. The infection assay with the mutant was performed in parallel with the non-mutated Siglec-10 as described above. As shown in Fig. 4(a, c), the mutation resulted in decreased infection, with a comparable infection rate and virus production to that seen in the control group (only expressing CD163). These results provide further evidence that the sialic acid-binding site in the N-terminal domain of Siglec-10 is critical for the infection process.

### PK-15 cells allow PRRSV attachment and internalization upon expression of Siglec-10

**Fig. 5.**
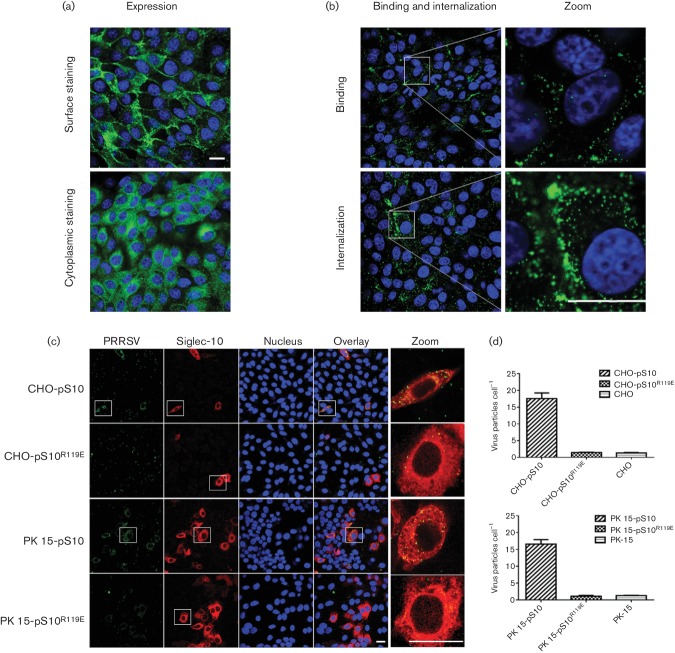
Porcine Siglec-10 mediates the endocytosis of PRRSV. (a) Immunofluorescence staining of Siglec-10 in stably transfected PK-15 ^S10+^ cells. PK-15 ^S10+^ cells were fixed with 4 % PF, and the cells were permeabilized (cytoplasmic staining) or not permeablized (surface staining) with 0.1 % Triton X-100 and stained with 1G10 mAb against Siglec-10 (green) and Hoechst 33 342 (nuclei; blue). Scale bar: 25 µm. (b) Attachment and internalization of PRRSV in PK-15 ^S10+^ cells. PK-15 expressing Siglec-10 or normal PK-15 cells were incubated with purified PRRSV LV for 1 h at 4 °C or 37 °C, allowing binding and internalization, respectively. After washing, the cells were fixed and stained with Hoechst 33 342 (nuclei; blue) and mAb 13E2 (PRRSV nucleocapsid protein; green), and analysed by confocal microscopy. Scale bar: 25 µm (c) CHO and PK-15 cells were transiently transfected with wild-type Siglec-10 or the Siglec-10^R119E^ mutant, and a virus internalization assay was performed 48 h post-transfection. Double staining for Siglec-10/Siglec-10^R119E^ (red) and co-localized PRRSV particles (13E2; green) was performed and analysed by confocal microscopy. Scale bar: 25 µm. (d) Quantification of PRRSV internalization in CHO and PK-15 cells for three independent experiments.

Previously, it was shown that PK-15 cells allow PRRSV attachment and internalization upon expression of pSn [8, 9]. To further analyse the specific function of Siglec-10 in the infection process of PRRSV, a cell line expressing Siglec-10 was established. Positive cell clones were further identified by IFA using antibodies against both the V5-tag and Siglec-10. Clones that were 100 % positive against both the V5 tag and Siglec-10 were selected (data not shown). Siglec-10 was present in the cytoplasm and on the plasma membrane (Fig. 5a). The stably transfected cell line was used for the binding and internalization assay. Upon the incubation of cells with viruses at 4 °C, an abundant number of virus particles were bound to the surface of the cells. Upon incubation at 37 °C, large numbers of virus particles were clustered both on the cell surface and inside the cytoplasm (Fig. 5b). For normal PK-15 cells, only a few virus particles could be observed on the surface of cells, possibly because of the presence of heparan sulfate [8], and no internalization was observed. To quantify the internalized particles and with that further prove the critical role of the sialic acid-binding site, the internalization assay for transient transfected PK-15 and CHO cells expressing wild-type Siglec-10 or the Siglec-10^R119E^ mutant were analysed and compared. A clear staining for internalized virus particles was observed in both cell types (PK-15 and CHO) expressing wild-type Siglec-10, but for the non-transfected cells or cells expressing Siglec-10^R119E^, only a few particles were detected at the plasma membrane of the cells (Fig. 5c, d). These results show that, similarly to Siglec-1, Siglec-10 is important for the attachment and internalization of PRRSV 23].

### Distribution of Siglec-10-positive cells in the porcine spleen

**Fig. 6.**
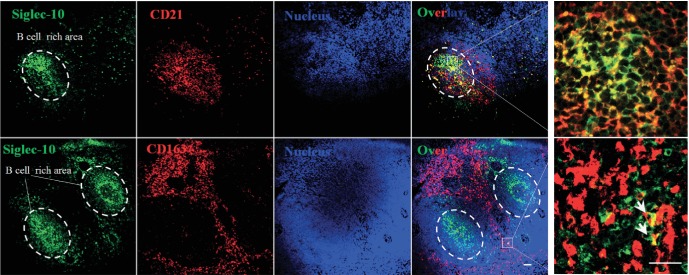
Immunofluorescence staining of Siglec-10/CD21 double-positive cells and Siglec-10/CD163 double-positive cells in tissue sections of the porcine spleen. Immunofluorescence staining of Siglec-10 and CD21 or Siglec-10 and CD163 in tissue sections of the porcine spleen. Tissue samples were sectioned (10 µm) and co-immunostained for Siglec-10 (green) and the markers CD21 (red) or CD163 (red). White dashed lines indicate the border between the B cell-rich area and CD163-rich area. White arrows show CD163^+^ Siglec-10^+^ double-positive cells. Scale bar: 25 µm.

Two areas of the porcine spleen were analysed, the B cell (CD21^+^)-rich area and the CD163-positive cell-rich area. Siglec-10 positive cells were mainly located in the centre of B cell-rich areas (100 % of the CD21-positive cells), but were also present in CD163^+^ cell-rich areas (16 % of the CD163^+^ cells) (Fig. 6). These results show that the majority of Siglec-10-positive cells in the spleen are B cells, and that a subset of monocytes that are Siglec-10-positive also exists.

## Discussion

Sn and CD163 are two key entry mediators for PRRSV. However, recent studies in our laboratory demonstrated that certain virus strains are able to infect Sn-negative cells [14]. Sn belongs to the Siglec family, containing members that are commonly used as receptors for various pathogens [19, 24, 25]. In this study, Siglec-10, a new Sn-like receptor, was identified as an additional binding and entry receptor for PRRSV. Siglec-10 was able to mediate the sialic acid-dependent binding of human erythrocytes and functioned in a similar way to Siglec-1 during PRRSV infection. However, clear differences were observed between the two PRRSV strains LV (type 1) and MN-184 (type 2).

Much Siglec research has been performed in humans. All Siglecs are type-1 membrane proteins that contain a Sia-binding, an amino-terminal V-set domain and varying numbers of C2-set Ig-like domains. As shown in Fig. 1(a), the predicted structures for porcine Siglec-3, Siglec-5 and Siglec-10 were quite similar. All of them showed only one V-set domain followed by 16, 1, 3 and 3 C2-type Ig domains for Siglec-1, Siglec-3, Siglec-5 and Siglec-10, respectively (Fig. 1a). The V-set domain and the adjacent C2-set domain contained a small number of invariant amino acid residues, including an ‘essential’ arginine on the F β-strand, as indicated in the structure with red dots and marked in the sequence alignment with an asterisk (Fig. 1a, b). This site has been predicted to be required for sialic acid binding in humans. In this study, the estimated binding site of Siglec-10 was mutated for verification of the sialic acid binding ability of Siglec-10. The sequences for Siglec-3, Siglec-5 and Siglec-10 obtained in our study were compared with those previously characterized by Alvarez and Escalona [20–22]. A high similarity was observed between the sequences, which demonstrates the high conservation of these Siglecs among pigs.

Siglecs are cell-surface proteins that bind sialic acids. In this study, both Siglec-1 and Siglec-10 showed strong red blood cell binding, but only after neuraminidase treatment. Most Siglecs are masked because of cis-interactions with sialic acids expressed on the cell surface. Following treatment with sialidase, Siglecs become unmasked, which allows them to interact with other ligands [16]. Binding was not observed in Siglec-3-expressing PK-15 cells even after treatment, which is in accordance with the studies by Alvarez and Escalona [20, 21]. For Siglec-5, only a few cells were detected that bound red blood cells in the three repeats. This is in contrast with the report from Escalona, who used purified protein for the porcine red blood cell binding assay [22]. Several factors might contribute to the observed differences. Firstly, for the purified protein, more abundant protein may be present for capturing the red blood cells, which may account for the higher binding capacity. Secondly, different production cell types were used in the two experiments, hence the expression and even the structure might be different, despite the close similarity of the sequences. Thirdly, the red blood cells used were different, which might also have contributed to the observed differences [22].

Previous research showed that sialoadhesin and CD163 join forces during the entry of PRRSV [7]. Because Siglec-1 is not necessary for infection with certain PRRSV strains [13, 26], we tried to find other Siglecs (Siglec-3, Siglec-5 and Siglec-10) that might have the same functionality as Siglec-1. Infection assays were performed with a non-permissive cell line that expressed the recombinant receptors. PK-15 cells were transfected with a pCD163-encoding plasmid, alone or in combination with a Siglec-1, Siglec-3, Siglec-5, Siglec-10 or Siglec-10^R119E^ mutant-encoding plasmid. After transfection, cells were treated or mock-treated with sialidase and then inoculated with the PRRSV type 1 LV strain and the PRRSV type 2 MN-184 strain. As it has previously been shown that MN-184 shows a higher cell tropism for Sn^-^ cells [26], the MN-184 strain was selected for the present study. The results showed that in addition to Siglec-1, Siglec-10 significantly increases the infection for both virus strains, although to a greater extent for MN-184. Based on the sequence analysis of the amino-terminal V-set domain, Siglec-10 shares a relatively high similarity with Siglec-1 [27].

In the present study, a correlation was found between red blood cell binding and PRRSV binding and infection. Indeed, the strong red blood cell binding of Siglec-1 and Siglec-10 coincided with improved PRRSV infection, whereas the weak red blood cell binding Siglec-5 showed only a minor increase in PRRSV infection, and the non-red blood cell binding Siglec-3 did not support PRRSV to any extent during infection.

PRRSV displays remarkable genetic, antigenic and clinical variability, resulting in two distinct groups of strains within the same viral family: type 1 (European type) and type 2 (American type) [3, 28]. Therefore, two strains, one of each type, were tested for their infectivity. Upon inoculation with LV, the number of infected cells and the level of virus production in cells expressing either Siglec-1 or Siglec-10 in combination with CD163 were significantly higher than in cells expressing only CD163 or cells expressing Siglec-3 or Siglec-5 in combination with CD163. Sialidase treatment of cells results in the removal of sialic acids on the cell surface, which allows more abundant binding of sialic acid-carrying particles such as human red blood cells and PRRSV. The treatment resulted in higher infection, indicating that Siglec-10 mediates virus entry in a sialic acid-dependent manner. With the MN-184 strain, a higher level of infection was observed compared to LV. Both Siglec-1 and Siglec-10 were able to improve the infection rate and virus production to almost the same level, regardless of sialidase treatment. It has been stated that the LV strain has a strict cell tropism for Sn^+^ macrophages [14, 29], whereas MN-184 and some other type 2 PRRSV strains are able to infect Sn^−^ cells [26]. In addition to Sn, these viruses most likely use another binding and entry receptor. Meanwhile, the most recent report from Yuste *et al*. [30] showed that PRRSV is able to replicate efficiently in splenic CD163^+^ macrophages that express low levels of Siglec-1 but high levels of Siglec-3 and Siglec-5. However, Siglec-3 and Siglec-5 did not seem to play a role in infection, which further confirmed our results showing that the non-permissive cell line expressing Siglec-3 or Siglec-5 in combination with CD163 did not improve virus infection and production. The results of the present study suggest that Siglec-10 might be a new receptor candidate for PRRSV binding/internalization, especially for type 2 viruses.

As mentioned earlier, an ‘essential’ arginine residue in all the known Siglecs is important for binding Sia-containing ligands. To further elucidate the function of Siglec-10, a site-directed mutation was performed. The predicted sialic acid-binding site aa 119 in the V-set domain was mutated from R to E. This resulted in a loss of human red blood cell binding activity and the absence of increased virus production. These results provide further evidence showing that the N terminal sialic acid-binding site is essential for virus infection, which is similar to Siglec-1 [31, 32]. Since sialoadhesin requires the sialic acid-binding activity to mediate the attachment of PRRSV [32], and given the observations above, we speculated that Siglec-10 might have a similar function during virus infection. A cell line expressing Siglec-10 was established and the virus binding and internalization assay was performed. The results showed that Siglec-10 was able to bind and internalize virus particles. In contrast, the cells expressing mutant Siglec-10^R119E^ were unable to bind and internalize the virus. Together, these results support the hypothesis that the sialic acid-binding site in the N terminal of Siglec-10 is crucial for virus binding and internalization (Fig. 5).

A previous study by Escalona *et al.* [21] showed that Siglec-10 was mainly expressed on B cells and also showed weak expression on monocytes. To check for the presence of splenic CD163^+^ macrophages expressing Siglec-10, double stainings for CD21/Siglec-10 and CD163/Siglec-10 were performed in the spleen. As was shown in the results, in the B cell-rich centre almost 100 % of the Siglec-10-positive cells were found to be CD21-positive, confirming the results from the work by Escalona *et al.* [21], in which it was seen that Siglec-10 is mainly expressed on B cells. Furthermore, in the CD163-positive area around 16 % of the CD163-positive cells co-expressed Siglec-10 (Fig. 6). Inconsistent reports exist regarding the expression of Siglec-10 in humans. Munday *et al.* [27] reported low levels of Siglec-10 expression on human CD19^+^ B lymphocytes and monocytes for a small subset of CD16^+^ CD56^-^ NK cells, and even lower levels on eosinophils. In contrast, Whitney *et al.* [33] reported no expression on B cells, whereas the granulocytes were Siglec-10-positive in humans [33]. The reactivity of the antibody used and the presence of different splicing variants of Siglec-10 might account for this variation. Based on the present study and a previous study by Escalona *et al.* [21], it can be stated that in pigs porcine Siglec-10 is mainly expressed on B cells and also on a minor subset of monocytes. The minor subset of monocytes may be an important new replication target for PRRSV *in vivo*. In addition, B-cells can be expected to act as a carrier for the virus *in vivo,* given that Siglec-10 is able to bind and internalize PRRSV particles. The absence of CD163 in B cells hampers PRRSV in infecting these cells. Siglec-10/-G has also been reported to be an inhibitory receptor on B cells [34–36]. Inhibitory receptors are known to influence various functions of immune cells, such as the regulation of cellular signalling, cell-to-cell interactions and endocytosis through an ITIM motif [16]. The sequence of porcine Siglec-10 contains one ITIM and one ITIM-like motif. When ITIM-possessing inhibitory receptors interact with their ligand, their ITIM motif becomes phosphorylated, allowing them to recruit other enzymes, such as SHP-1 and SHP-2. These kinds of phosphatases will decrease the activation of the molecules involved in cell signalling [37], which can negatively regulate signal transduction [38]. Human Siglec-10 is reported to be associated with the tyrosine phosphatase SHP-1, a known negative regulator of nuclear factor κB (NF-κB) activation [39], while the inhibition of NF-κB activation is mediated by SHP-1 via the ITIM motif of Siglec-10 [33]. NF-κB belongs to a family of inducible transcription factors that are involved in pathogen- or cytokine-induced immune and inflammatory responses, as well as cell proliferation and survival [40]. One of the most remarkable features of PRRSV infection is the failure to elicit the expression of inflammatory cytokines in the lungs of pigs, particularly type I interferons, interleukin-1 (IL-1) and tumour necrosis factor alpha (TNF-α), which are important in antiviral responses. Whether this phenomenon is related to the function of Siglec-10 needs to be identified [41, 42]. In our study, in addition to its binding and internalization ability, there is a possibility that Siglec-10 also acts as an inhibitory immune receptor to facilitate the infection of PRRSV. Further studies are needed to test the hypothesis that PRRSV can interact with Siglec-10 via the ITIM motif on B cells, leading to downregulation of immune-related signal transduction, and therefore possibly escape the immune system.

In conclusion, this study revealed that, similarly to Siglec-1, Siglec-10 is able to improve PRRSV infection in non-permissive cells in combination with CD163. Like Siglec-1, Siglec-10 is able to mediate the attachment and endocytosis of PRRSV, which is dependent on the sialic acid-binding activity of the N-terminal immunoglobulin domain. Siglec-10 showed a higher affinity towards the type 2 PRRSV strain MN-184compared to the type 1 PRRSV strain LV. For the type 2 PRRSV strain MN-184, Siglec-10 was as performant as Siglec-1 in its receptor function, whereas for the type 1 PRRSV strain LV, Siglec-1 was the most effective entry mediator. In the future, more work will be required to determine the replication kinetics of other PRRSV strains in Siglec-10-positive cells, and the impact of PRRSV replication in these cells on the immune response.

## Methods

### Cell lines, viruses and antibodies

PK-15 (porcine kidney) cells were grown in MEM supplemented with 10 % foetal bovine serum (FBS) and a mixture of antibiotics. Marc-145 cells were cultivated as described previously [7]. They were then maintained in a humidified 5 % CO_2_ atmosphere at 37 °C. The European prototype PRRSV LV strain was passaged 13 times on macrophages and subsequently 5 times on Marc-145 cells. The MN-184 virus strain (American type) was passaged five times on Marc-145 cells.

PRRS virions were visualized via the nucleocapsid protein specific mAb 13E2 [26, 43] and a secondary conjugate. For detection of the V5-tag, a mouse monoclonal antibody (GenScript; A00641) and a goat anti-mouse IgG horseradish peroxidase (HRP)-labelled secondary antibody (Dako; P0447) were used, or a directly labelled mAb conjugated with FITC (Invitrogen; R963-25), was used. For the visualization of CD163, either a mouse monoclonal antibody (2A10/11, IgG1; AbD Serotec, Dusseldorf, Germany) or a goat polyclonal antibody (R and D Systems, Minneapolis) with appropriate secondary conjugates were used.

### Cloning, construction and identification of porcine Siglecs

The primer pairs were designed based on the sequences submitted and characterized on NCBI (Table 1). The coding region of putative porcine Siglecs were amplified and cloned. Briefly, total RNA was extracted from porcine spleen (Siglec-10) or PBMCs (Siglec-3 and Siglec-5) using the RNeasy mini kit (Qiagen). Afterwards, reverse-transcription PCR (RT-PCR) was performed using random primers with the SuperScript III reverse transcriptase kit according to the standard procedure (Thermo Science). After RT-PCR amplification with the Herculase DNA polymerase kit (Agilent Technologies), the purified PCR product was cloned into the pCDNA3.1D/V5-His-TOPO vector in frame with the V5 tag (Invitrogen) via restriction enzymes and T4 ligase (Invitrogen). Constructs were verified by sequencing. Porcine sialoadhesin and CD163 were previously cloned into the pcDNA3.1D/V5-His-TOPO vector (Invitrogen) and the pBudCE4.1 vector (Invitrogen) [7, 9]. The spatial structure of the Siglecs was predicted using the I-TASSER online tool (https://zhanglab.ccmb.med.umich.edu) and analysed by PyMOL (version 6.6). Comparisons of the predicted domains, motifs and features of Siglecs from different species were performed on the Sample Modular Architecture Research Tool (SMART). For further functional analysis of Siglec-10, a point-mutation (R119E) was introduced into the predicted sialic acid-binding domain of Siglec-10 with the primers pS10RE-FW and pS10RE-RV, as listed in Table 1. Site-directed mutagenesis was carried out using the Quick-change site-directed mutagenesis kit (Agilent Technologies) according to the manufacturer’s instructions.

**Table 1.** The list of primers used in the study Primers used for PCR amplification of Siglec-3, -5 and -10 and for the mutagenesis of Siglec-10, artificial restriction sites (underlined) for cloning, mutated residues (bold)

**Name**	**Primer Sequence**	**Fragment size**	**Purpose**
Siglec3-FL-F	5′ -GTT*AAGCTT*aGCCACCATGCGGCCGCTGCTGCTGCT-3′	1143 bp	Full-length cloning of Siglec-3
Siglec3-FL-R	5′-TCTt*CTCGAG*ttCCGGGTCCCGATCTCTGTGTAT-3′		
Siglec5-FL-F	5′-GTT*AAGCTT*GCCACCATGGTGCCCCTGCTGCTGCTGCTG-3′	1665 bp	Full-length cloning of Siglec-5
Siglec5-FL-R	5′-TCT*CTCGAG*ttTTTGCTTTTTCTGATCTCTGAGTAC-3′		
Siglec10-FL-F	5′-CTT*GGTACC*GCCACCATGCTCCTGCCGCTGCTCTTAG-3′	1842 bp	Full-length cloning of Siglec-10
Siglec10-FL-R	5′-GCT*GCGGCCGC*TGTGGAACTGGACCGCAGCATATT-3′		
pS10^R119E^-FW	5′-CATGCCGCCTACTTCTTT**gaa**TTGGAGAGAGGCCCTTAC-3′	1842 bp	Mutation of binding site for Siglec-10
pS10^R119E^-RV	5′-GTACGGCGGATGAAGAAA**ctt**AACCTCTCTCCGGGAATG-3′		

### Expression analysis of Siglecs in eukaryotic cells by immunofluorescence analysis and Western blot

PK-15 cells were transiently transfected with the Siglec-encoding vectors using Lipofectamine (Invitrogen) following the manufacturer’s instructions. Forty-eight hour post-transfection, the cells were fixed with 4 % paraformaldehyde, permeabilized with 0.01 % Triton X-100 or not permeabilized, and stained with Hoechst (10 µg ml^−1^; Invitrogen) and directly labelled V5-FITC mAbs (1 : 500, Invitrogen; R963-25). The results were analysed by (confocal) fluorescence microscopy (Leica Microsystems GmbH, Heidelberg, Germany).

For Western blot analysis 48 h post-transfection, cells were collected and washed using ice-cold phosphate-buffered saline (PBS). Cellular lysates were prepared by lysis in buffer containing 50 mM Tris (pH 7.4), 150 mM NaCl, 1 % Nonidet P-40, 0, 1 % SDS, 5 mM EDTA and protease inhibitor cocktail (Roche) in water. Samples were either left untreated, or treated with endoglycosidase H, PNGase F (New England Biolabs, Inc.) or 100 mU/ml *Vibrio cholerae* sialidase (Roche Applied Science) for 3 h at 37 °C. Afterwards, samples were mixed with reducing Laemli buffer (5×) SDS-PAGE loading dye, boiled for 10 min, and subjected to SDS-PAGE (12 % gel) using a BioRad Mini Protean 3 system. Proteins were transferred to a PVDF membrane (Membrane Hybond-P; GE Healthcare) using a BioRad mini trans-blot system, and then the membranes were blocked overnight using blocking solution (5 % skimmed milk in PBS, 0.1 % Tween-20). A V5-specific mAb (GenScript; A00641) diluted 1 : 2000 in PBS and peroxidase-labelled goat anti-mouse IgG antibodies (Dako) diluted 1 : 1000 in blocking solution were used for the detection of protein. HRP-conjugated anti-alpha tubulin monoclonal antibody (Abcam, ab40742) was used to stain tubulin. The results were visualized with an ECL Western blotting detection system (GE Healthcare).

### Red blood cell binding assay

Human red blood cells were obtained from healthy donors and stored at 4 °C in Alsever’s solution. After three washings, cells were diluted in RPMI. PK-15 cells were transiently transfected with the Siglec-encoding vectors using Lipofectamine (Invitrogen). Forty-eight hours post transfection, cells were washed with RPMI and half of the wells were pre-treated with 10 mU/ml *Vibrio cholerae* sialidase (Roche) in RPMI for 1 h at 37 °C. The other half were mock-treated with RPMI. After treatment, cells were washed three times with RPMI and incubated with washed erythrocytes (0.25 % v/v in RPMI) for 1 h at 37 °C. Subsequently, cells were washed and erythrocyte binding was evaluated by light microscopy.

### Infection experiments on non-target cells expressing recombinant receptors with or without neuraminidase treatment

PK-15 cells were transiently transfected with a pCD163-encoding vector alone (pCD163^+^) or in combination with a Siglec-encoding vector (pCD163^+^ Siglec^+^). Forty-eight hours post-transfection, cells were washed and pre-treated with 10 mU/ml *Vibrio cholerae* sialidase (Roche) in RPMI or just mock-treated with RPMI for 1 h at 37 °C. After three washes with PBS, cells were inoculated with 250 µl PRRSV LV or MN-184 virus at a m.o.i. of 0.5. At 1 h p.i., the inoculum was removed, cells were washed three times with PBS and washing fluids were replaced with 300 µl MEM containing 10 % FCS. At 24 h p.i., cell supernatants were collected and cells were fixed with ice-cold methanol. For the quantification of infected cells, fixed cells were incubated with the PRRSV N-specific monoclonal antibody 13E2 (IgG2a) followed by a secondary goat anti-mouse IgG2a FITC-labelled antibody (Invitrogen) [43]. To reduce the background signal, 10 % negative goat serum was included for blocking during each step. Cell nuclei were stained with Hoechst (10 µg ml^−1^, Invitrogen) for 10 min at room temperature. The infection level and expression of the different Siglecs and pCD163 were quantified by confocal microscopy (absolute number of virus- or receptor-positive cells). To determine the titre of extracellular virus, the collected supernatant was centrifuged to remove cell debris and used for virus titration. For titration on Marc-145 cells, cells were seeded 3 days before inoculation. Monolayers were inoculated with a 10-fold dilution series of the samples and incubated for 7 days at 37 °C. The cytopathic effect (CPE) was then visualized by light microscopy. Finally, the virus titres were calculated as TCID_50_ ml^−1^[44]. Parallel experiments were performed for the binding site-directed mutation construct.

### PK-15 ^S10+^ cell line establishment, virus binding and internalization analysis

PK15 cells were transfected with the Siglec-10-encoding plasmid containing the geneticin resistance gene using Lipofectamine (Invitrogen). The Siglec-10-expressing PK15 cells were selected with geneticin (200 µg ml^−1^, GIBCO) and subsequently subcloned. Cells were initially identified using immunofluorescence staining, primarily using V5-FITC mAbs (Invitrogen; R963-25) and further confirmed using a monoclonal antibody (1G10) against Siglec-10 that was developed in this study. To stain the PK-15^S10+^ cells, cells were fixed with 4 % PF and permeabilized or not permeabilized with 0.1 % Triton X-100 for cytoplasmic staining or surface staining, respectively. Next, cells were incubated with 1G10 (IgG1) diluted in PBS containing 10 % NGS, followed by an incubation with secondary goat anti-mouse IgG1 FITC-labelled antibody. MAb 13D12 was used as an isotype-matched irrelevant control [45]. Cell nuclei were stained with Hoechst for 10 min at room temperature. The established cell line was used for the virus binding and internalization assay. For virus binding, cells were inoculated with purified LV virus at 4 °C for 1 h. Then, cells were fixed with 4 % paraformaldehyde. For internalization, the cells were fixed with 4 % paraformaldehyde 1 h post-virus inoculation. After fixation, cells were washed with PBS and permeabilized with a 0.1 % Triton X-100 solution in PBS. As a control, non-transfected cells were also fixed after inoculation of the virus, and subsequently washed and permeabilized. Cells were stained for PRRSV particles using 13E2 specific to PRRSV N protein [43] and goat anti-mouse FITC antibody. The antibody 1C11 against gB of PrV (IgG2a) was used as an irrelevant isotype control [45]. The results were analysed by confocal microscopy (Leica Microsystems GmbH).

For quantification of the internalized virus particles for cells expressing Siglec-10, PK-15 and CHO cells were transiently transfected with Siglec-10 and Siglec-10^R119E^ mutant expressing plasmid with Lipofectamine. Cells were inoculated with purified PRRSV LV at a m.o.i. of 5, and after incubation for 1 h at 37 °C, cells were washed and fixed with methanol. Virus particles were stained using PRRSV nucleocapsid-specific mAb 13E2 (IgG 2a), and Siglec-10 was stained using 1G10 (1 : 10, IgG1). The antibodies 13D12 against gD of PRV (IgG1) and 1C11 against gB of PrV (IgG2a) were used as irrelevant isotype controls [45]. Co-localization of Siglec-10 with virus particles was counted (15 cells for each experimental condition).

### Monoclonal antibody production

Five 4–6 week-old Balb/c mice were immunized intramuscularly with 2 µg of recombinant Siglec-10-encoding eukaryotic expression plasmid and boosted 2 and 4 weeks later with the same amount. After four immunizations, all of the mice had seroconverted. Serum was collected from immunized mice and used as a source of polyclonal antibodies. The mouse with the highest antibody titre was selected for a final boost with PK-15^S10+^ cells. Four days later, spleen lymphocytes were fused with the SP2/0 myeloma cells as described previously [43, 46], using polyethylene glycol 4000 (Sigma). Siglec-10-specific hybridomas were screened by performing a cell-based IPMA with the hybridoma supernatant. Briefly, PK-15^S10+^ cells were fixed by drying and subsequent incubation with 4 % paraformaldehyde and methanol +1 % H_2_O_2_. Next, cells were incubated with undiluted hybridoma supernatant for 1 h at 37 °C, followed by biotinylated anti-mouse IgG antibody and streptavidin-biotinylated HRP complex (GE healthcare). Afterwards, AEC substrate was added and the results were analysed by light microscopy. Positive hybridomas were subcloned by limiting dilution. The isotype of the obtained mAb was determined by ELISA with a mouse monoclonal antibody isotyping test kit from Zymed Laboratories, Inc.

### Immunoﬂuorescence staining analysis for Siglec-10 in porcine spleen tissue sections

To identify Siglec-10-positive cells in tissues, 10 µm thick cryosections of frozen porcine spleen tissues were made and fixed in 100 % methanol at −20 °C for 15 min. Porcine spleen tissues were collected from three conventional pigs. For double staining of B cells and Siglec-10 positive cells, primary mouse monoclonal antibodies against the B cell marker CD21 (IgG2b) and Siglec-10 (1G10, IgG1) and isotype-specific secondary antibodies conjugated with FITC or Alexa Fluor 594 (Invitrogen) were used. Cell nuclei were stained with Hoechst 33 342. For the staining of CD163 and Siglec-10 double-positive cells, goat polyclonal antibody against CD163 (R and D Systems, Minneapolis) and mouse polyclonal antibody against Siglec-10 were incubated at 4 °C overnight. After three washes with PBS, sections were incubated with rabbit anti-goat AF594 for 1 h at 37 °C. Then, the sections were blocked with negative rabbit serum for 30 min and incubated with goat anti-mouse biotin-labelled secondary antibody, followed by streptavidin FITC-labelled antibody. For the staining with biotin-labelled antibody, the tissues were pre-treated with an avidin/biotin blocking kit (Thermo Fisher). Counting was performed in 3 cryosections for each pig, with 10 fields per cryosection, selected in a random way. Results were analysed using a Leica TCS SPE laser-scanning confocal microscope (Leica Microsystems GmbH). Positive cells were counted within regions of interest (ROIs), including the B cell-rich area and the CD163-rich area, and calculated using Image J.

### Statistical analysis

All experiments were performed three times. Statistical significance (*P<0.05; **P<0.01; ***P<0.001) was calculated using the two-way ANOVA test followed by the Bonferroni post-test to determine the differences between the different receptor-transfected groups and the control group, and also the treated and untreated groups. All of the statistical analyses were performed using Graphpad Prism 5.
